# Use of explicit ICD9-CM codes to identify adult severe sepsis: impacts on epidemiological estimates

**DOI:** 10.1186/s13054-016-1497-9

**Published:** 2016-10-03

**Authors:** C. Bouza, T. Lopez-Cuadrado, J. M. Amate-Blanco

**Affiliations:** 1 Health-Care Technology Assessment Agency, Institute of Health Carlos III, Madrid, Spain; 2 National Centre of Epidemiology, Institute of Health Carlos III, Madrid, Spain

**Keywords:** Severe sepsis, Epidemiology, Health services research, Incidence, Outcome, Trends

## Abstract

**Background:**

Severe sepsis is a challenge for healthcare systems, and epidemiological studies are essential to assess its burden and trends. However, there is no consensus on which coding strategy should be used to reliably identify severe sepsis. This study assesses the use of explicit codes to define severe sepsis and the impacts of this on the incidence and in-hospital mortality rates.

**Methods:**

We examined episodes of severe sepsis in adults aged ≥18 years registered in the 2006–2011 national hospital discharge database, identified in an exclusive manner by two ICD-9-CM coding strategies: (1) those assigned explicit ICD-9-CM codes (995.92, 785.52); and (2) those assigned combined ICD-9-CM infection and organ dysfunction codes according to modified Martin criteria. The coding strategies were compared in terms of the populations they defined and their relative implementation. Trends were assessed using Joinpoint regression models and expressed as annual percentage change (APC).

**Results:**

Of 222 846 episodes of severe sepsis identified, 138 517 (62.2 %) were assigned explicit codes and 84 329 (37.8 %) combination codes; incidence rates were 60.6 and 36.9 cases per 100 000 inhabitants, respectively. Despite similar demographic characteristics, cases identified by explicit codes involved fewer comorbidities, fewer registered pathogens, greater extent of organ dysfunction (two or more organs affected in 60 % versus 26 % of cases) and higher in-hospital mortality (54.5 % versus 29 %; risk ratio 1.86, 95 % CI 1.83, 1.88). Between 2006 and 2011, explicit codes were increasingly implemented. Standardised incidence rates in this cohort increased over time with an APC of 12.3 % (95 % CI 4.4, 20.8); in the combination code cohort, rates increased by 3.8 % (95 % CI 1.3, 6.3). A decreasing trend in mortality was observed in both cohorts though the APC was −8.1 % (95 % CI −10.4, −5.7) in the combination code cohort and −3.5 % (95 % CI −3.9, −3.2) in the explicit code cohort.

**Conclusions:**

Our findings suggest greater and increasing use of explicit codes for adult severe sepsis in Spain. This trend will have substantial impacts on epidemiological estimates, because these codes capture cases featuring greater organ dysfunction and in-hospital mortality.

**Electronic supplementary material:**

The online version of this article (doi:10.1186/s13054-016-1497-9) contains supplementary material, which is available to authorized users.

## Background

Adult severe sepsis is associated with high mortality, morbidity and hospital costs [1–5] and remains a real challenge for clinicians and health care systems [5–8]. Population estimates of severe sepsis and their trends are essential to assess disease burden and estimate healthcare resource requirements [3, 8–10].

Owing to the difficulty in prospectively identifying cases at the population scale [8–11], population-based studies of severe sepsis have been based on the *International Classification of Diseases, Ninth Edition, Clinical Modification* (ICD-9-CM). However, reported incidence estimates of severe sepsis and related hospital mortality vary widely from 13 to 300 cases per 100,000 inhabitants and from 28 % to 50 %, respectively [1, 2, 4, 9]. Among other factors, this disparity seems to be determined by biases introduced by the different strategies used to identify cases [9, 12, 13].

Following the definition in 1991 of severe sepsis as sepsis associated with acute organ dysfunction [14], population estimates of the incidence of severe sepsis and its associated mortality have been based on combination algorithms of ICD-9-CM infection and organ dysfunction codes [15, 16]. Recognizing the limitations of such algorithms, sometimes described as excessively inclusive and with scarce specificity to represent real cases of severe sepsis [10, 16, 17], in 2002, a set of explicit ICD-9-CM codes was issued [18]. However, despite this coding system having been developed more than 10 years ago, and the specificity being close to 100 % [19], studies conducted in the USA indicate that the use of this strategy is scarce and is restricted to patients with more severe sepsis [13]. In effect, both for population-based and other research studies, combination codes are most widely used [9, 13]. However, it is not known if this practice is generalized because no European population-based studies have addressed the use of explicit severe sepsis codes, and the impacts of the given coding system used on epidemiological incidence and mortality estimates are unknown.

The present study sought to identify cases of severe sepsis captured by explicit and combination codes from a national registry, and compare these two coding strategies in terms of: (1) their implementation to identify severe sepsis in adults in Spain and their impacts on incidence estimates and (2) their definition of a given profile of patient demographics, clinical characteristics and hospital outcomes.

## Methods

### Design and data sources

We performed a retrospective study using the official clinical-administrative database designated National Minimum Basic Data Set (MBDS) of the Spanish Ministry of Health, Social Services and Equality (MSSSI). In the Spanish national health system, when a patient is discharged from hospital, the responsible physician is required by law to record all diagnoses and clinical procedures performed using the ICD-9-CM system. This information is compiled in the MBDS database. This database is considered to be representative of the national population as it includes data on over 90 % of all hospitalizations in the country annually [19, 20].

In the MBDS, each hospitalization is treated as a specific record and includes information on patient demographics, type of admission, date of admission, date of discharge, destiny upon discharge, along with diagnosis codes including the principal diagnosis, 13 secondary diagnoses and up to 20 procedures performed during hospitalization. Hospitalization data for the study period were obtained from the MSSSI [19] and data for the general population were obtained from the National Statistics Institute (Instituto Nacional de Estadística) [21].

### Study population: case identification and definitions

We identified all hospitalizations of adult patients (≥18 years) with severe sepsis from 1 January 2006 to 31 December 2011. To capture all cases, we used two established ICD-9-CM diagnostic coding strategies [22–24], generating two cohorts of longitudinal data. The first strategy was based on explicit ICD-9-CM codes (995.92 for severe sepsis, 785.52 for septic shock) [9, 13, 18, 22] and the second on ICD-9-CM infection and organ dysfunction combination codes according to modified Martin criteria. This second strategy is detailed in Additional file 1: Table S1. In addition, the codes are given for septicaemia, fungaemia and bacteraemia as described by Martin et al. [16, 22]. This strategy includes the ICD-9-CM code for sepsis (995.91) introduced in Spain in 2004. The two sets of codes were assigned in a mutually exclusive fashion.

The codes defining organ dysfunction are provided in Additional file 2: Table S2. The choice of this combination strategy was based on studies indicating its capacity to estimate the burden of severe sepsis [23] and the fact that it has been used in a previous study by our group [24]. This provided us with two cohorts of longitudinal data designated the explicit codes cohort and the combination codes cohort.

We assessed the registry data for the 13 secondary diagnosis fields using the version of the Charlson index validated by Deyo for ICD-9-CM [25], to assess comorbidities [26]. This index includes specific comorbid conditions of known prognostic value, which are classified using ICD-9 codes from prior outpatient and in-patient codes. Prior epidemiological studies in sepsis have shown that there is no overlap between the codes used to calculate this index and the diagnostic codes used to capture acute organ dysfunction [27], and that it is useful in assessing the risk of death in septic patients [28]. For identification of specific microorganisms, code 041 was included as indicated by the ICD-9-CM coding manual for the purpose of identifying bacterial agents in the case of diseases classified under the heading “other” [20].

### Ethics

All data were anonymized. According to Spanish legislation the use of these data is exempt of the need for informed consent [29].

### Data analysis

A descriptive comparative analysis was performed to compare the use of explicit and combination coding practices for severe sepsis, including data on patient demographics, comorbidities, organ dysfunction and in-hospital mortality. In addition, in the explicit codes cohort, differentiation was made between hospitalizations coded 785.52 (septic shock) and those coded 995.92 (severe sepsis).

The Charlson-Deyo index was calculated according to the improved STATA 14 package, both as a continuous variable and as a categorical variable with four groups (score 0, 1–2, 3–4 and >4) of increasing severity and impact on outcome [30]. In-hospital mortality was calculated as the number of deaths divided by the number of cases of severe sepsis in each cohort and expressed as a percentage.

Qualitative variables were expressed as absolute frequencies and percentages and quantitative variables as means and standard deviations. Association between qualitative variables was assessed using Pearson's chi-squared test or Fisher's exact test, and between quantitative and qualitative variables using Student's *t* test. We used the risk ratio (RR) with its respective 95 % confidence interval to quantify differences in demographic and clinical data between the cohorts.

Incidence rates were estimated using national data for subjects aged ≥18 years expressed as results per 100,000 inhabitants. Age-adjusted incidence and in-hospital mortality rates were calculated by the direct standardization method using the year 2008 as reference. To identify trends in incidence and in-hospital mortality rates, we quantified the annual percentage change (APC) with its respective 95 % confidence interval, using log-linear regression models assuming a standard Poisson distribution [24, 31]. This procedure serves to determine whether an apparent change in trend is statistically significant using a Monte Carlo permutation method [31]. All statistical tests were performed using the programmes STATA 14 (© 1985–2015 StataCorp LP. TX 77845 USA) and Joinpoint Regression (version 4.2.0.2, 23 June 2015). Significance was set at *p* < 0.05.

## Results

Over the six-year period, there were 222 846 adult hospitalizations for severe sepsis. The codes used for these hospitalizations were combination in 84 329 (37.8 %) and explicit in 138 517 (62.2 %). Among the latter, 93 380 cases (67 %) were coded 785.52 and 45 137 (33 %) were coded 995.92.

### General characteristics

Both the explicit code and combination code cohorts predominantly comprised men (Table 1) and mean age was similar (71 years, *p* = 0.120). In contrast, hospitalizations coded using explicit codes comprised a lower comorbidity burden (mean Charlson index 2 vs. 2.2, *p* < 0.001) and a lower percentage of cases in the categories of greater severity. Further, with the exception of neoplasms, the frequencies of each of the comorbidities included in the Charlson index were also lower in this cohort than in the combination code cohort. There were more surgical cases among the patients assigned explicit codes.

**Table 1:** General characteristics and in-hospital mortality recorded in the two cohorts examined (n = 222 846)

	Explicit codes cohort n = 138 517 (62.2)	Combination codes cohort n = 84 329 (37.8)	RR (95 % CI)
Men	80 102 (57.8)	49 730 (59.0)	0.98 (0.97, 0.99)
Age groups (years)			
18–44	10 355 (7.5)	7093 (8.4)	0.89 (0.86, 0.92)
45–64	30 129 (21.8)	17 187 (20.4)	1.07 (1.05, 1.09)
65–74	29 375 (21.2)	17 159 (20.4)	1.04 (1.02, 1.06)
75–84	44 348 (32.0)	26 801 (31.8)	1.01 (0.99, 1.02)
> 84	24 310 (17.6)	16 089 (19.1)	0.92 (0.90, 0.94)
Categorized Charlson index			
0	32 843(23.7)	15 884 (18.8)	1.26 (1.24, 1.28)
1–2	63 820 (46.1)	37 781 (44.8)	1.03 (1.02, 1.04)
3–4	26 796 (19.3)	20 730 (24.6)	0.79 (0.77, 0.80)
> 4	15 058 (10.9)	9934 (11.8)	0.92 (0.90, 0.95)
Specific comorbidities^a^			
Diabetes	29 243 (21.1)	20 207 (24.0)	0.88 (0.87, 0.90)
Cancer	28 064 (20.3)	15 321 (18.2)	1.12 (1.09, 1.14)
Chronic heart failure	23 053 (16.6)	14 024 (16.6)	1.00 (0.98, 1.02)
COPD	20 624 (14.9)	14 934 (17.7)	0.84 (0.82, 0.86)
Chronic renal disease	18 275 (13.2)	17 660 (20.9)	0.63 (0.62, 0.64)
Liver disease	17 408 (12.6)	10 616 (12.6)	1.00 (0.97, 1.02)
Cerebrovascular disease	10 394 (7.5)	8089 (9.6)	0.78 (0.76, 0.81)
Dementia	8139 (5.9)	5354 (6.3)	0.93 (0.89, 0.96)
Peripheral vascular disease	7515 (5.4)	4441 (5.3)	1.03(0.99, 1.07)
Acute myocardial infarction	5587 (4.0)	3800 (4.5)	0.90 (0.86, 0.93)
Surgical pathology	41 518 (30.0)	17 378 (20.6)	1.45 (1.43, 1.48)
Microbiological data^a^			
Pathogens identified:	52 946 (38.2)	52 641 (62.4)	0.61 (0.60, 0.62)
Gram-positive bacteria	21 591 (40.8)	25 804 (49.0)	0.83 (0.82, 0.85)
Gram-negative bacteria	36 765 (69.4)	30 944 (58.8)	1.18 (1.16, 1.20)
Fungi	1521 (2.9)	2525 (4.8)	0.60 (0.56, 0.64)
Site of infection^a^			
Respiratory system	35 176 (25.4)	18 572 (22.0)	1.15 (1.13, 1.17)
Genitourinary tract	28 517 (20.6)	22 507 (26.7)	0.77 (0.76, 0.78)
Abdomen	16 178 (11.7)	4414 (5.2)	2.23 (2.16, 2.31)
Central nervous system	1425 (1.0)	1082 (1.3)	0.80 (0.74, 0.87)
Procedure-related	13 608 (9.8)	9848 (11.7)	0.84 (0.82, 0.86)
Soft tissue	5285 (3.8)	2842 (3.4)	1.13 (1.08, 1.19)
Others/not specified	52 838 (38.2)	19 593 (23.2)	1.64 (1.62, 1.67)
Organ dysfunction (number of organs)			
1	44 614 (32.2)	62 371 (74.0)	0.44 (0.43, 0.44)
2	41 994 (30.3)	16 956 (20.1)	1.51 (1.48, 1.54)
> 2	42 222 (30.5)	5002 (5.9)	5.14 (4.99, 5.30)
Not specified^b^	9687 (7.0)	0	Not applicable
Type of organ system dysfunction^a^			
Cardiovascular	97 135 (70.1)	6081 (7.2)	9.72 (9.5, 10.0)
Respiratory	68 680 (49.6)	39 679 (47.0)	1.05 (1.04, 1.07)
Renal	60 192 (43.5)	35 552 (42.2)	1.03 (1.02, 1.04)
Haematological	17 300 (12.5)	8743 (10.4)	1.20 (1.17, 1.24)
Metabolic	13 396 (9.7)	5409 (6.4)	1.51 (1.46, 1.56)
Neurological	11 232 (8.1)	12 525 (14.9)	0.55 (0.53, 0.56)
Hepatic	7687 (5.6)	4529 (5.4)	1.03 (0.99, 1.07)
Invasive therapeutic measures			
Mechanical ventilation	38 450 (27.8)	13 353 (15.8)	1.75 (1.71, 1.79)
Haemodialysis	11 699 (8.5)	7847 (9.3)	0.91 (0.88, 0.93)
In-hospital death	75 495 (54.5)	24 758 (29.4)	1.86 (1.83, 1.88)

Data presented as number of cases (%). ^a^Subgroups not mutually exclusive; ^b^organ dysfunction without number of organs specified. *RR* risk ratio, *CI* 95 confidence interval, *COPD* chronic obstructive pulmonary disease

Microorganisms were recorded in a significantly lower proportion of cases in the explicit code cohort. Gram-negative bacteria were the most frequently reported. As potential sources of sepsis, the abdomen, respiratory tract and soft tissues were significantly more frequently recorded in this cohort than in the combination code cohort. The possible source was not specified in 38 % of the explicit code cohort and in 23 % of the combination code cohort.

The extent of organ dysfunction differed significantly between the two cohorts (Table 1). Thus, in the explicit code group, a third of all episodes featured the dysfunction of one, two, or more than two organs, while in the combination code cohort, 74 % of cases involved single-organ dysfunction. When comparing affected organs, cardiovascular, respiratory, kidney, haematological and metabolic dysfunction were significantly more frequently recorded in the explicit code cohort, as was the use of invasive mechanical ventilation. It should be noted that no data were available on the number or type of organ dysfunction in 7 % of cases in the explicit code cohort.

Table 2 shows the characteristics of the cases recorded in the explicit code cohort according to whether they were coded 785.52 (septic shock) or 995.92 (severe sepsis). In this cohort, 67 % of cases were coded 785.52 and corresponded to younger patients with fewer comorbidities and a greater number of organ dysfunctions, i.e., cardiovascular, haematological, metabolic and respiratory, and a greater need for mechanical ventilation support. However, it should be noted that in a large proportion of cases codified as 995.92, data were not available on the number or type of dysfunctional organs. Gram-negative microorganisms and respiratory sources of infection were largely recorded in both groups.

**Table 2:** Characteristics of cases identified using explicit codes (n = 138 517)

	Code 785.52^a^ n = 93 380 (67 %)	Code 995.92^a^ n = 45 137 (33 %)	RR (95 % CI)
Men	54 906 (58.8)	25 196 (55.8)	1.05 (1.04, 1.07)
Age group (years)			
18–44	7434 (8.0)	2921 (6.5)	1.23 (1.18, 1.28)
45–64	22 135 (23.7)	7994 (17.7)	1.34 (1.30, 1.37)
65–74	21 025 (22.5)	8350 (18.5)	1.22 (1.19, 1.25)
75–84	29 374 (31.4)	14 974 (33.2)	0.95 (0.93, 0.97)
> 84	13 412 (14.4)	10 898 (24.1)	0.59 (0.58, 0.61)
Charlson comorbidity index			
0	23 482 (25.2)	9361 (20.7)	1.21 (1.18, 1.24)
1–2	43 180 (46.2)	20 640 (45.7)	1.01 (0.99, 1.03)
3–4	17 029 (18.2)	9767 (21.6)	0.84 (0.82, 0.86)
> 4	9689 (10.4)	5369 (11.9)	0.87 (0.84, 0.90)
Detailed comorbidities^b^			
Diabetes	18 184 (19.5)	11 059 (24.5)	0.79 (0.78, 0.81)
Cancer	19 671 (21.1)	8393 (18.6)	1.13 (1.10, 1.16)
Chronic heart failure	13 892 (14.9)	9161 (20.3)	0.73 (0.71, 0.75)
COPD	13 488 (14.4)	7136 (15.8)	0.91 (0.89, 0.94)
Chronic renal disease	11 327 (12.1)	6948 (15.4)	0.79 (0.76, 0.81)
Liver disease	11 922 (12.8)	5486 (12.1)	1.05 (1.02, 1.08)
Cerebrovascular disease	6155 (6.6)	4239 (9.4)	0.70 (0.67, 0.73)
Dementia	4237 (4.5)	3902 (8.6)	0.52 (0.50, 0.55)
Peripheral vascular disease	4726 (5.1)	2789 (6.2)	0.82 (0.78, 0.86)
Acute myocardial infarction	3690 (4.0)	1897 (4.2)	0.94 (0.89, 0.99)
Surgical pathology	30 850 (33.0)	10 668 (23.6)	1.40 (1.37, 1.43)
Microbiological data			
Identified pathogens^b^	35 418 (37.9)	17 528 (38.8)	0.98 (0.96, 1.0)
Gram-positive pathogens	14 318 (40.4)	7273 (41.5)	0.97 (0.95, 1.01)
Gram-negative pathogens	24 639 (69.6)	12 126 (69.2)	1.00 (0.98, 1.03)
Fungi	1043 (2.9)	478 (2.7)	1.08 (0.97, 1.21)
Site of infection^b^			
Respiratory system	24 476 (26.2)	10 700 (23.7)	1.11 (1.08, 1.13)
Genitourinary tract	17 462 (18.7)	11 055 (24.5)	0.76 (0.75, 0.78)
Abdomen	12 294 (13.2)	3884 (8.6)	1.53 (1.47, 1.58)
Procedure- related	9448 (10.1)	4160 (9.2)	1.10 (1.06, 1.14)
Soft tissue	3483 (3.7)	1802 (4.0)	0.93 (0.88, 0.99)
Central nervous system	991 (1.1)	434 (1.0)	1.10 (0.98, 1.24)
Cardiovascular system	884 (1.0)	398 (0.9)	1.07 (0.95, 1.21)
Others/not specified	35 257 (37.8)	17 581 (39.0)	0.97 (0.95, 0.99)
Organ system dysfunction (number of organs)			
1	27 222 (29.1)	17 392 (38.5)	0.76 (0.74, 0.77)
2	30 393 (32.6)	11 601 (25.7)	1.26 (1.24, 1.29)
> 2	35 765 (38.3)	6457 (14.3)	2.68 (2.61, 2.75)
Not specified^c^	Not applicable	9687 (21.5)	Not applicable
Type of organ system dysfunction^b^			
Cardiovascular	93 380 (100)	3755 (8.3)	12.0 (11.63, 12.42)
Respiratory	47 490 (50.9)	21 190 (47.0)	1.08 (1.07, 1.10)
Renal	38 789 (41.5)	21 403 (47.4)	0.88 (0.86, 0.89)
Haematological	12 220 (13.1)	5080 (11.3)	1.16 (1.13, 1.20)
Metabolic	9418 (10.1)	3978 (8.8)	1.14 (1.10, 1.19)
Neurological	7083 (7.6)	4149 (9.2)	0.83 (0.79, 0.86)
Hepatic	5197 (5.6)	2490 (5.5)	1.00 (0.96, 1.06)
Invasive therapeutic measures			
Mechanical ventilation	30 569 (32.7)	7881 (17.5)	1.88 (1.83, 1.92)
Haemodialysis	9052 (9.7)	2647 (5.9)	1.65 (1.58, 1.73)
In-hospital death	50 993 (54.6)	24 502 (54.3)	1.00 (0.99, 1.02)

Data presented as number of cases (%). ^a^Code 785.52: septic shock, code 995.92: severe sepsis; ^b^subgroups not mutually exclusive; ^c^organ dysfunction without number of organs specified. *RR* risk ratio, *CI* confidence interval, *COPD* chronic obstructive pulmonary disease

## Trends in the use of explicit or combination codes for severe sepsis

**Fig. 1:**
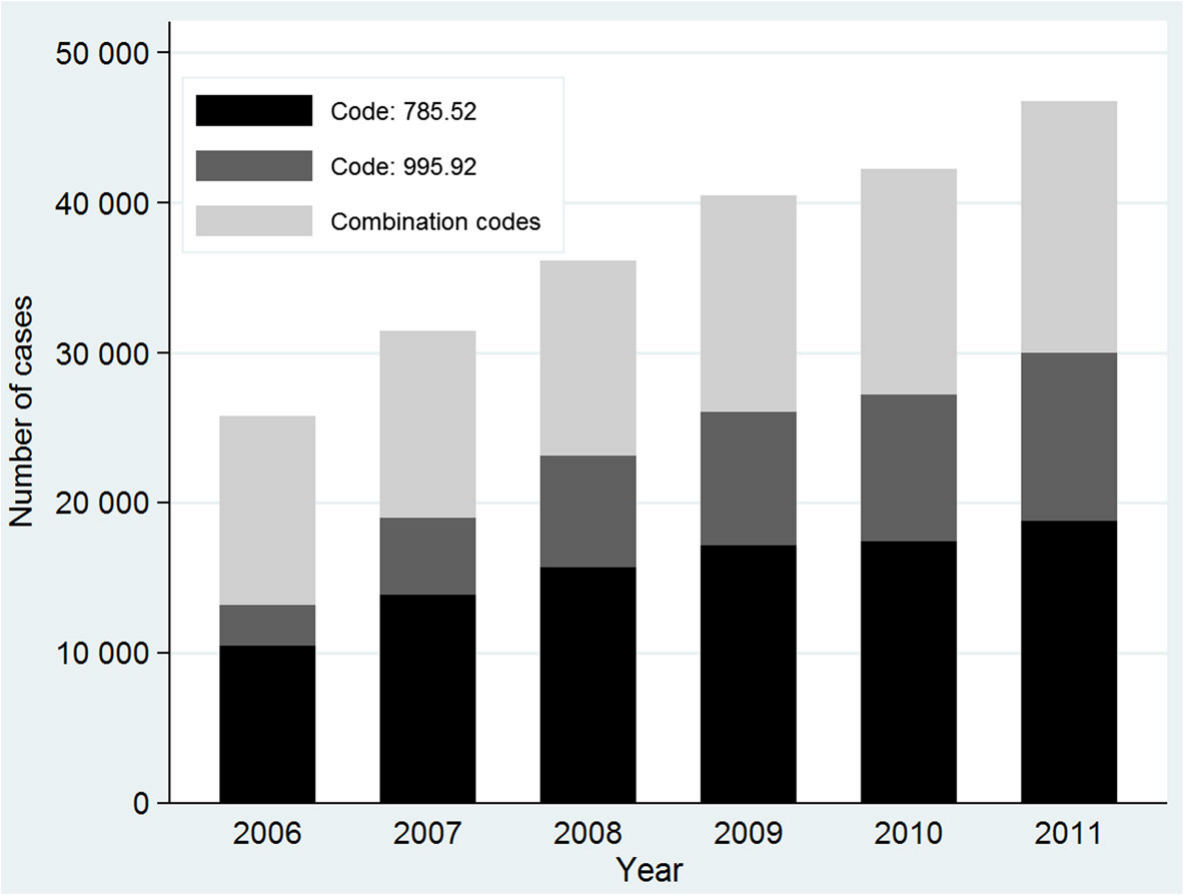
Cases of severe sepsis identified using explicit and combination codes across the study interval. From 2006 to 2011, the number of cases rose from 12 652 to 16 748 in the combination code cohort and from 13 156 to 30 026 in the explicit code cohort. Within this cohort, cases coded 995.92 (severe sepsis) rose from 2697 to 11 233 and those coded 785.52 (septic shock) rose from 10 459 to 18 793

In 2006, 51 % and 49 % of cases were coded using explicit and combination codes respectively. However, in the last year of the study period (2011), these figures were 64.2 % and 35.8 % respectively. Trends in explicit coding practices (Fig. 1) indicate the fairly stable use of code 785.52 (from 40.5 % to 40.2 % across the period) and a notable increase in cases coded 995.92 (from 10.4 % in 2006 to 24 % in 2011).

## Incidence

The episodes of severe sepsis identified amounted to 1.1 % of all adult hospitalizations over the 6-year period and an overall incidence of 97.5 cases per 100,000 inhabitants. However, crude incidence rates were 36.9 cases per 100 000 inhabitants for the combination codes and 60.6 cases per 100,000 inhabitants for the explicit codes. In this cohort, incidence rates were 19.75 cases per 100,000 inhabitants for code 995.92 (severe sepsis) and 40.85 cases per 100 000 inhabitants for code 785.52 (septic shock).

**Fig. 2:**
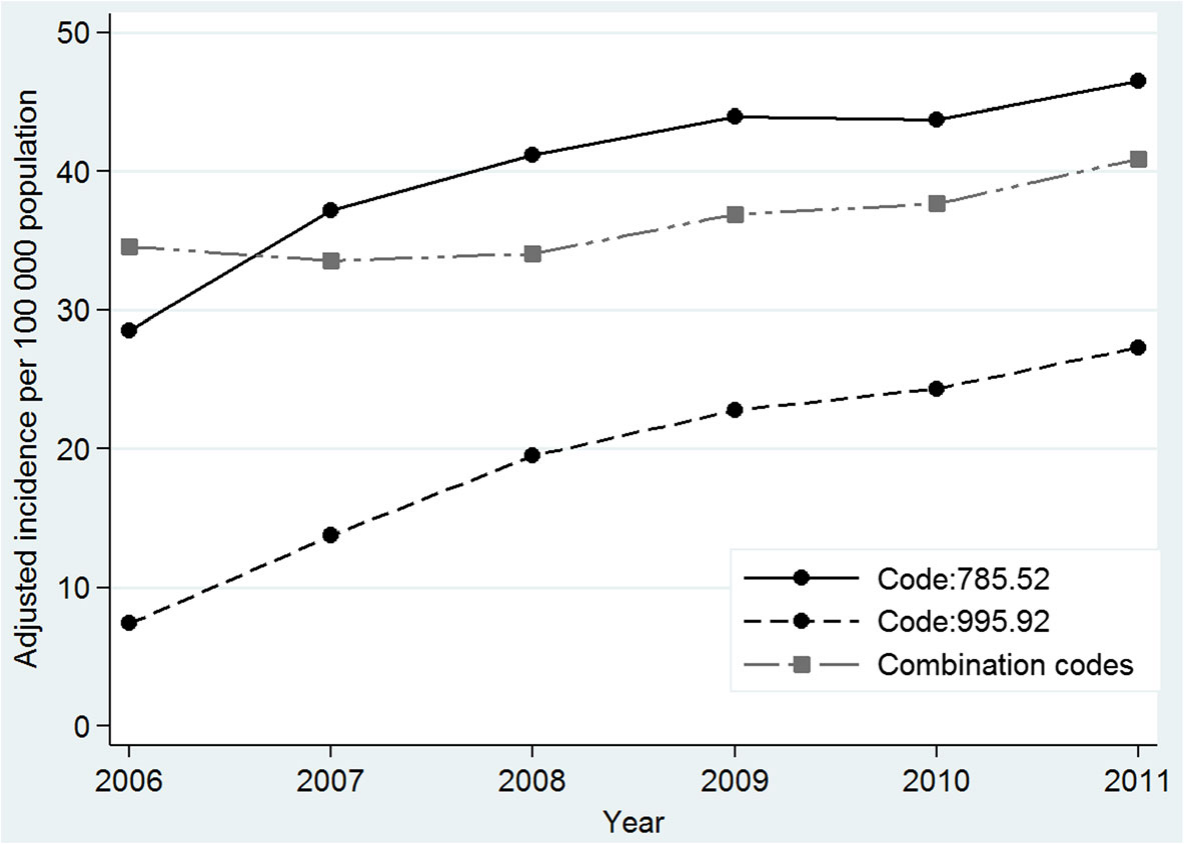
Trends in age-adjusted incidence of severe sepsis according to assigned ICD-9-CM codes. Values are adjusted annual rates. In the explicit code cohort, adjusted incidence rates increased from 36 cases per 100 000 inhabitants in 2006 to 73.6 per 100 000 in 2011, and from 34.6 to 40.9 cases per 100,000 inhabitants in the combination code cohort. Within the cohort of explicit codes, the adjusted incidence rate of cases assigned code 785.52 (septic shock) rose from 28.6 to 46.5 cases per 100 000 inhabitants, while cases identified using 995.92 (severe sepsis) rose from 7.4 to 27.3 cases per 100 000 inhabitants

From 2006 to 2011, the overall number of captured cases increased from 25 808 to 46 774, representing an annual increase of 13.5 %. Figure 1 shows the number of cases of severe sepsis identified using explicit and combination codes across the study interval. In the explicit code cohort, adjusted incidence rates (Fig. 2) went from 36 cases per 100 000 inhabitants in 2006 to 73.6 per 100 000 in 2011, giving an APC of 12.3 % (95 % CI 4.4, 20.8) and from 34.6 to 40.9 cases per 100 000 inhabitants in the combination code cohort, giving an APC of 3.8 % (95 % CI 1.3, 6.3). Within the cohort of explicit codes, the adjusted incidence rate of cases assigned code 785.52 increased from 28.6 to 46.5 cases per 100 000 inhabitants, giving an APC of 8.1 % (95 % CI 2.2, 14.3), while cases identified using 995.92 changed from 7.4 to 27.3 cases per 100 000 giving an APC of 21.7 % (95 % CI 7.2, 38.1).

### Mortality

Overall in-hospital mortality was 45 % (n = 100 253 cases) while 29.4 % was recorded for the combination code cohort and 54.5 % was recorded for the explicit code cohort. In the explicit code cohort, 39 % of deaths (n = 29 310) were produced in critical care units, while this figure was 20 % (n = 4962) in the combination code cohort.

As may be observed in Table 2, in the cohort of explicit codes, mortality rates were similar for cases captured using codes 995.92 or 785.52 (54.3 % vs. 54.6 %).

**Fig. 3:**
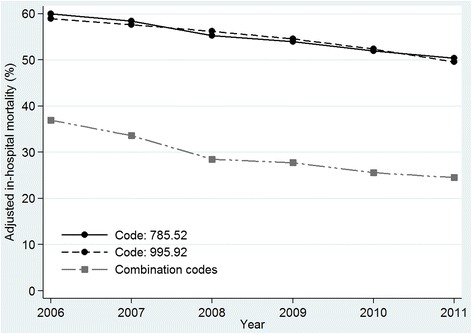
Age-adjusted in-hospital mortality rate for severe sepsis according to discharge ICD-9-CM codes. Values are adjusted annual mortality rates. From 2006 to 2011, in-hospital mortality had a significantly decreasing trend in both cohorts. In the combination code cohort in-hospital mortality fell from 37 % in 2006 to 25 % in 2011. In the explicit code cohort, in-hospital mortality fell from 60 % in 2006 to 50 % in 2011. Within the explicit cohort, rates over the same period diminished from 60 % to 50.4 % in the code 785.52 group (septic shock) and from 59 % to 49.6 % in the code 995.92 group (severe sepsis)

From 2006 to 2011, the adjusted in-hospital mortality rate had a significantly decreasing trend in both cohorts. However, the combination code cohort dropped, with an APC of −8.1 % (95 % CI −10.4, −5.7 %) while the decline, though significant, was less pronounced in the explicit code cohort with an APC over the study period of −3.5 % (95 % CI −3.9, −3.2). Figure 3 shows the changes detected in each cohort and explicit sub-cohorts. In the code 785.52 group, mortality rates diminished, with an APC of −3.5 % (95 % CI −4.0, –3.0). The 995.92 sub-cohort had a similar decrease, with an APC of −3.5 % (95 % CI −4.4, –2.6).

**Fig. 4:**
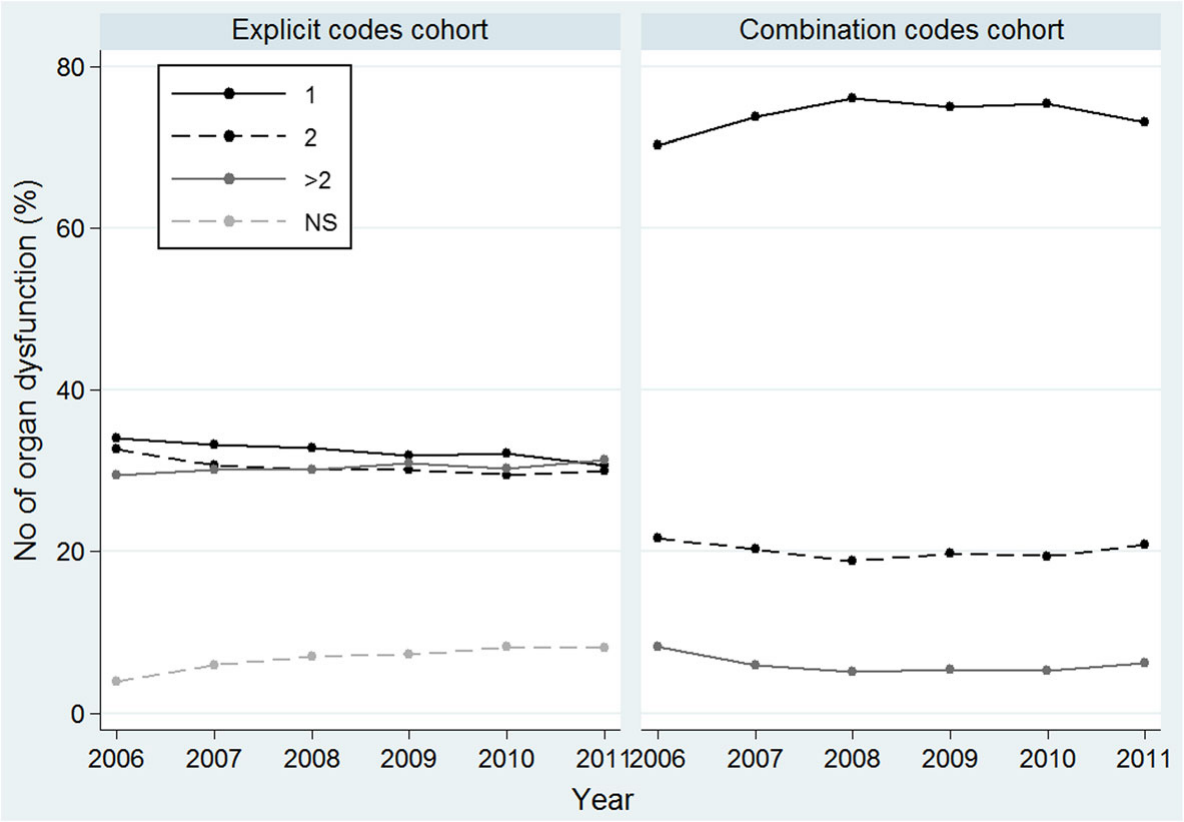
Trends in the number of organ dysfunctions recorded in the two cohorts examined. Values are percentages. In the explicit code cohort, 34 % of cases involved single organ dysfunction, 32.7 % two organs and 29.4 % more than two affected organs in 2006 versus 30.7 %, 30.2 % and 31.3 %, respectively in 2011. In 2006, the number of dysfunctional organs was not specified in 3.9 % compared with 8 % in 2011. In the combination code cohort, 70.3 % of cases involved single organ dysfunction, 21.6 % two affected organs and 8.4 % more than two affected organs in 2006 versus 73.1 %, 20.8 % and 6.1 %, respectively in 2011

Figure 4 shows that the trend in the extent of organ dysfunction was stable over the study period in both cohorts. However, we detected an increase in the percentage of cases in which the number of organ dysfunctions was not recorded in the explicit code cohort.

## Discussion

The results of this study reveal the elevated use of explicit codes to define severe sepsis in Spain and an upward trend in this practice over the period 2006 to 2011. Compared to combination severe sepsis codes, these ICD-9-CM codes captured a case profile featuring greater organ dysfunction, healthcare effort and in-hospital mortality. These differences will have an enormous impact on estimates of disease burden. Our findings also indicate that cases coded with explicit 785.52 (septic shock) or 995.92 (severe sepsis) codes, though having different characteristics, had similar outcomes and practically the same in-hospital mortality.

Our findings are inconsistent with recent reports from the USA such as that by Gaieski et al. [9], who in a retrospective study based on nationwide in-patient data for 2004 to 2009, found that only a minority of cases of severe sepsis (between 14 % and 36.9 % according to the capture algorithm used) were assigned explicit discharge codes. In another retrospective study by Whittaker et al. conducted at a single tertiary hospital [13], it was observed that among 1735 cases of severe sepsis between 2005 and 2009, only 21.5 % of cases had explicit severe sepsis/septic shock discharge codes (995.92, 785.52) and that this trend remained stable over the period examined. In contrast, in our study over 60 % of cases in the national health network were documented with such codes. In addition, the implementation of these explicit codes, especially code 995.92, had an increasing trend over the six-year study period. Although the reasons for this difference are unknown, given Spain's universal health system, we can assume that coding practices will not be related to financial incentives. With regards to other factors, it is likely that the efforts to improve coding strategies for hospital discharge registries and the education programmes and campaigns carried out in our country in recent years, such as the Edusepsis campaign [32], will have improved the awareness and training of healthcare professionals in the identification and diagnosis of severe sepsis, leading to the observed increased use of explicit codes.

In line with prior findings [15, 16, 22], we detected a marked increase in the incidence of severe sepsis though, notably, this increase was mainly accounted for by the cohort of explicit codes, and especially, of code 995.92. Even if the interpretation of this increase may be confounded in part by factors such as better diagnosis of sepsis, improvement in coding practices or other methodological issues [33], the specificity of these codes [9] suggests that the rising trend in the incidence of severe sepsis in adults in our country may be real and not the consequence of excessive coding of infection and organ dysfunction [23, 34].

In addition, although the populations included in both our cohorts should have been similar as the codes assigned define the same disease, the patients in each cohort had different outcomes and were only well-matched in terms of age and sex.

In agreement with another report [13], our data indicate that it was the cases of greater severity that were assigned explicit codes, and that these codes defined a cohort of patients who, despite having fewer comorbidities, had a higher rate of infection of pulmonary and abdominal origin, more Gram-negative pathogens, and above all, more affected organs and a higher mortality rate. In-patient mortality for the cohort captured using explicit codes was 54 % and practically doubled the rate recorded for the cohort identified through combination codes. Notably, mortality in this latter cohort was similar to the rates reported in other population studies, such as those of Angus [15] and Martin [16], in which combination strategies were exclusively used to identify cases, while our rate for the explicit code cohort was consistent with the mortality rates cited for intensive care units [35, 36]. Furthermore, in this cohort we recorded practically identical mortality rates among cases coded 995.92 or 785.52. While there are appreciable differences in the demographics, comorbidities and potential infection sources in these cases, both groups featured similar multiple organ dysfunction and this factor likely accounts for the high mortality observed in both sub-cohorts [37].

Recent data indicate there is great variability in mortality due to severe sepsis and septic shock which, among other factors, seems to be related to the different definitions used in each study [38]. In addition, studies assessing the use of codes 785.52 (septic shock) and 995.92 (severe sepsis) have been scarce. On reanalysis, Gaiesky [9] observed that hospital mortality among adults with explicit codes was 36.9 % for those coded 995.92 and 42.2 % for those coded 785.52. In 373 patients coded as having severe sepsis/septic shock (995.92, 785.52), Whittaker [13] observed 28-day mortality of 41 %. However, no study has compared the epidemiological characteristics and progression among cases coded 995.92 and 785.52. We feel the novelty of our work is that this type of comparison was performed using a national population database including a large number of cases and a representative case-mix. Very probably, because of the new sepsis definitions [39] in which the use of explicit codes is recommended, new studies with which to compare our findings will soon emerge.

The high percentage of cases assigned explicit codes and their high mortality rate, in large measure explains the differences observed in in-hospital mortality between the present and other population studies including a lower percentage of cases captured by explicit severe sepsis codes. Further, although mortality in our study had an overall declining trend from 2006 to 2011, this decline was significantly lower in the explicit code cohort. Thus, although our data reflect the decreasing trend in hospital mortality due to severe sepsis in adult patients observed in other studies [9, 16, 40, 41], it remains clear that this trend is more limited in cases of greater disease severity. Interestingly, in both our cohorts a stable trend in the extent of organ dysfunction was produced, which despite not confirming recently published data from the USA [34], could nevertheless explain, at least partly, the observed downward trend in mortality.

The elevated proportion of hospitalizations assigned explicit codes observed in our study is perfectly in line with the new definitions of sepsis and recommendations for the use of ICD-9 codes 995.92 and 785.52 [39] for such cases. In effect, although the implementation of these explicit codes is not yet complete in Spain, it is still high and shows an increasing trend. Consistent with these recommendations [39], we predict that better understanding of the concept and definition of severe sepsis through continued education programmes will improve its description in clinical records and thus allow for a more consistent measure of the burden of severe sepsis and its trends.

The limitations of our study are those inherent to investigations based on retrospectively collected clinical-administrative data. Although there are national directives for the use of the ICD-9-CM coding system, this may not have been uniform across all hospitals of the national health network and we cannot rule out coding errors despite regular audits making major errors unlikely. We are also aware that, because of its confidential nature, the database used lacks sufficient information and the data do not allow for causal inferences. However, the use of such databases is well-established in severe sepsis epidemiology and the results of a recent meta-analysis clearly support the use of administrative data to monitor mortality trends in severe sepsis [41], confirming the essential role of the consistent use of national administrative data for epidemiological monitoring of incidence and outcomes [9, 10].

Our country has a large national population-based database covering over 90 % of all hospitalizations produced annually in the country. There are potential benefits of this system including representativeness, identification of systemic problems, and precision of estimation in statistical analysis. Additionally, this study followed the publication guidelines for observational studies laid down in the strengthening the reporting of observational studies in epidemiology (STROBE) initiative [42].

A limitation of our study was that non-hospitalized patients with severe sepsis were not included, meaning that incidence was really an estimate of treated sepsis [43]. Similarly, our mortality estimates were conservative in that we did not include mortality after hospital discharge [3, 5, 44].

## Conclusions

Our study reveals an elevated and increasing use of explicit coding practices for adult severe sepsis in Spain. This trend will have substantial impacts on epidemiological and disease burden estimates, because cases are registered of greater severity, care intensity and in-hospital mortality. The variation detected in severe sepsis coding and its effects on population estimates calls for improved continuous education for physicians and the introduction of standardized measures targeted at reducing heterogeneity in coding practices.

## Key messages

- In Spain over the period 2006–2011, some 62 % of adult severe sepsis cases in this population-based study were assigned explicit ICD-9-CM codes.
- This elevated and increasing use of explicit codes for adult severe sepsis has substantial impacts on epidemiological estimates, because these codes capture a case profile featuring extensive organ dysfunction, care effort and in-hospital mortality.
- Variability in severe sepsis coding practices must be taken into account when interpreting epidemiological estimates.
- Inconsistencies in medical coding practices call for the implementation of sepsis education programmes for health professionals.

## Additional files

Additional file 1: Table S1. ICD-9-CM codes used to identify hospitalizations for severe sepsis in the combination codes cohort. (DOC 36 kb) Additional file 2: Table S2. ICD-9-CM codes used to define organ dysfunction. (DOC 31 kb)

## Abbreviations

APC: Annual percent change
COPD: Chronic obstructive pulmonary disease
ICD-9-CM: International Classification of Diseases, 9th revision, Clinical Modification
MBDS: National Minimum Basic Data Set
RR: Risk ratio
