# Supplementary material for: Use of explicit ICD9-CM codes to identify adult severe sepsis: impacts on epidemiological estimates
Source: Crit Care. 2016 Oct 3;20:313. doi: 10.1186/s13054-016-1497-9 (PMC5047045; doi:10.1186/s13054-016-1497-9)
Supplement: Additional file 1: Table S1. — ICD-9-CM codes used to identify hospitalizations for severe sepsis in the combination codes cohort. (DOC 36 kb) [file 13054_2016_1497_MOESM1_ESM.doc]

**Table S1. ICD-9 codes used to identify hospitalizations with severe sepsis in the combination codes cohort**

| **ICD9-CM codes** |  |
| --- | --- |
| 038.0 | Streptococcal septicaemia |
| 038.1 | Staphylococcal septicaemia |
| 038.2 | Pneumococcal septicaemia |
| 038.3 | Septicaemia due to anaerobes |
| 038.4 | Septicaemia due to other Gram negative organisms |
| 038.8 | Other specified septicaemias |
| 038.9 | Unspecified septicaemia |
| 003.1 | Salmonella septicaemia |
| 020.2 | Septicaemic plague |
| 036.2 | Meningococcal septicaemia |
| 036.3 | Waterhouse-Friderichsen syndrome |
| 054.5 | Herpetic septicaemia |
| 098.89 | Gonococcaemia |
| 112.5 | Systemic candidiasis |
| 112.81 | Candida endocarditis |
| 117.9 | Other and unspecified mycoses |
| 790.7 | Bacteraemia |
| 995.91 | Sepsis, systemic inflammatory response syndrome due to infectious process without organ dysfunction |
