# Supplementary material for: Use of explicit ICD9-CM codes to identify adult severe sepsis: impacts on epidemiological estimates
Source: Crit Care. 2016 Oct 3;20:313. doi: 10.1186/s13054-016-1497-9 (PMC5047045; doi:10.1186/s13054-016-1497-9)
Supplement: Additional file 2: Table S2. — ICD-9-CM codes used to define organ dysfunction. (DOC 31 kb) [file 13054_2016_1497_MOESM2_ESM.doc]

**Table S2. ICD-9-CM codes used to define organ dysfunction**

| **Organ dysfunction** | **ICD9-CM codes** |
| --- | --- |
| **Respiratory** | 518.81 (acute respiratory failure),  518.82 (other pulmonary insufficiency),  518.84 (acute on chronic respiratory failure),  518.85 (acute respiratory distress syndrome after shock or trauma),  786.09 (respiratory distress, insufficiency),  799.1 (respiratory arrest),  96.7 (invasive mechanical ventilation) |
| **Cardiovascular** | 785.5 with all sub codes (shock without  trauma, includes 785.1, 785.9),  458 (hypotension, 458.0, 458.8 458.9),  796.3 (nonspecific low blood pressure) |
| **Renal** | 584 with all sub codes (acute renal failure),  580 (acute glomerulonephritis),  39.95 (haemodialysis) |
| **Hepatic** | 570 (acute and subacute necrosis of liver),  572.2 (hepatic coma),  573.3 (hepatitis, unspecified) |
| **Haematologic** | 286.6 (defibrination syndrome),  286.9 (other and unspecified coagulation defects),  287.3-5 (secondary thrombocytopaenia, unspecified) |
| **Neurologic** | 293 (acute delirium),  348.1 (anoxic brain damage),  348.3 (encephalopathy, unspecified),  357.82 (critical illness polyneuropathy),  780.01 (coma),  780.09 (drowsiness, unconsciousness, stupor)  89.14 (electroencephalogram) |
| **Metabolic** | 276.2 (acidosis metabolic or lactic) |
